# Revealing tissue architecture through the hypercomplex Fourier analysis of spatial transcriptomics data

**DOI:** 10.1093/bioadv/vbaf191

**Published:** 2025-08-11

**Authors:** Hildreth Robert Frost

**Affiliations:** Biomedical Data Science, Dartmouth College, Hanover, NH 03755, United States

## Abstract

**Motivation:**

We present an approach for analyzing spatial transcriptomics (ST) data using a quaternion-domain discrete Fourier transform. Quaternions are four-dimensional hypercomplex numbers that have been primarily employed to represent rotations in computer graphics with biomedical applications focused on biomolecule structure and orientation.

**Results:**

According to our proposed model, the quaternion associated with each location in an ST dataset represents a vector in $R^{3}$ whose length captures sequencing depth and whose direction captures three transcriptomic features (individual genes, gene sets, or latent variables). This representation has several important benefits: (i) it enables the use of powerful Fourier-based image analysis techniques on a multidimensional representation of ST data, (ii) it implies that transformations in transcriptomic state can be viewed as three-dimensional rotations with a corresponding representation as rotation quaternions, and (iii) it supports an ST visualization that captures transcriptomic uncertainty. We demonstrate the features of this model through the analysis of Visium HD data and discuss how a similar model can be applied to single-cell RNA-sequencing data.

**Availability and implementation:**

An implementation of this model, support for the hypercomplex Fourier analysis of ST data, and example vignettes are included in the QSC R package (https://hrfrost.host.dartmouth.edu/QSC/).

## 1 Introduction

### 1.1 Quaternions

Quaternions are four-dimensional hypercomplex numbers that, along with real numbers, complex numbers and octonions, represent one of the four normed division algebras. Formally, hypercomplex numbers are elements of a finite-dimensional algebra over the real numbers that is unital but may not be associative or commutative (Kantor and Solodovnikov 1989) and have a general representation given by:


$$a_{0}+\sum_{j=1}^{n}a_{j}i_{j},i_{j}^{2}\in \{-1,0,1\}$$

where $a_{0}$ and $a_{j}$ are real numbers and the $i_{j}$ form an *n*-dimensional basis with $i_{j}^{2}$ usually choosen to equal −1. Following this representation, n=0 for real numbers, n=1 for complex numbers, n=3 for quaternions, and n=7 for octonions with $i_{j}^{2}=-1$, i.e. real numbers, complex numbers, quaternions, and octonions are the hypercomplex numbers with dimensions 1, 2, 4, and 8 that are each comprised by a real number and a vector in an *n*-dimensional imaginary space and, as proved by Frobenius, represent the only four normed division algebras. While real and complex numbers are commonly employed in applied mathematics to model experimental data, quaternions, and especially ontonions, are infrequently used outside of a theoretical context.

Quaternions were first explored by Gauss in 1809, however, because that work was not published until 1900, the discovery of quaternions is usually credited to the Irish mathematician William Hamilton in 1843 (1844). Hamilton’s discovery was motivated by attempts to extend complex numbers to three dimensions. In his work on three-dimensional hypercomplex numbers, Hamilton struggled with multiplication/division, i.e. what is the quotient of two points in $R^{3}$? Hamilton realized these problems could be solved by moving to four-dimensional hypercomplex numbers and, when he had this realization, famously carved the formula for the quaternion imaginary basis vectors into the Brougham Bridge:


$$i^{2}=j^{2}=k^{2}=\mathrm{ijk}=-1$$

More specifically, a quaternion *q* is a four-dimensional hypercomplex number defined by (1).


(1) q=a+bi+cj+dk

where {a,b,c,d}∈R and *i*, *j*, and *k* form a three-dimensional imaginary basis with:


(2) $$\begin{array}{c} i^{2}=j^{2}=k^{2}=\mathrm{ijk}=-1\\ ij=k,ji=-k,jk=i,kj=-i,ki=j,ik=-j \end{array}$$

Addition and subtraction of quaternions is simply performed by adding and subtracting the associated terms. For example, if $q_{1}=a_{1}+b_{1}i+c_{1}j+d_{1}k$ and $q_{2}=a_{2}+b_{2}i+c_{2}j+d_{2}k$, then $q_{1}+q_{2}=a_{1}+a_{2}+(b_{1}+b_{2})i+(c_{1}+c_{2})j+(d_{1}+d_{2})k$. Multiplication of quaternions follows the distributive law and the rules for multiplication of the basis vectors given by (2). An important consequence of (2) is that multiplication of quaternions is not commutative, e.g. ij≠ji. Division of quaternions is defined by the pre or post multiplication by the inverse of the denominator, which, given the noncommutativity of multiplication, may be distinct. So, $q_{1}/q_{2}$ has two possible values: $q_{1}q_{2}^{-1}$ or $q_{2}^{-1}q_{1}$. The inverse of a quaternion is defined as the ratio of the conjugate ($q^{*}$) and the square of the norm (||q||):


(3) $$q^{-1}=\frac{q^{*}}{||q|{|}^{2}}=\frac{a-bi-cj-dk}{a^{2}+b^{2}+c^{2}+d^{2}}$$

It is often convenient to separate quaternions into scalar (r=a) and vector ($\vec{v}=bi+cj+dk$) parts:


(4) $$q=a+bi+cj+dk=r+\vec{v}=(r,\vec{v})$$

If b=c=d=0, the quaternion is referred to as a scalar quaternion. If a=0, the quaternion is referred to as a vector or pure quaternion. Using the scalar/vector notation, multiplication of quaternions can be conveniently represented using vector dot and cross product notation:


(5) $$\begin{array}{c} q_{1}q_{2}=(r_{1},{\vec{v}}_{1})(r_{2},{\vec{v}}_{2})\\ =(r_{1}r_{2}-{\vec{v}}_{1}\cdot {\vec{v}}_{2},r_{1}{\vec{v}}_{2}+r_{2}{\vec{v}}_{1}+{\vec{v}}_{1}\times {\vec{v}}_{2}) \end{array}$$

Interpretation of (5) can be aided by considering two special cases:

1. Multiplication by a scalar: If $q_{2}$ is a scalar quaternion, i.e. ${\vec{v}}_{2}=0$, then the multiplication simplifies to the scaling of $q_{1}$ by the scalar part of $q_{2}$:
  $$\begin{array}{c} \left(r_{1}r_{2}-{\vec{v}}_{1}\cdot {\vec{v}}_{2},r_{1}{\vec{v}}_{2}+r_{2}{\vec{v}}_{1}+{\vec{v}}_{1}\times {\vec{v}}_{2}\right)\\ \left(r_{1}r_{2}-{\vec{v}}_{1}\cdot 0,r_{1}0+r_{2}{\vec{v}}_{1}+{\vec{v}}_{1}\times 0\right)\\ \left(r_{1}r_{2},r_{2}{\vec{v}}_{1}\right) \end{array}$$
2. Multiplication of two vector quaternions: If both $q_{1}$ and $q_{2}$ are vector quaternions, i.e. $r_{1}=r_{2}=0$, then multiplication simplifies to just the dot and cross product terms:
  $$\left(-{\vec{v}}_{1}\cdot {\vec{v}}_{2},{\vec{v}}_{1}\times {\vec{v}}_{2}\right)$$

If ${\vec{v}}_{1}⊥{\vec{v}}_{2}$, then $q_{1}q_{2}={\vec{v}}_{1}\times {\vec{v}}_{2}$, which is a vector quaternion that is orthogonal to the plain defined by $q_{1}$ and $q_{2}$. If ${\vec{v}}_{1}||{\vec{v}}_{2}$, then $q_{1}q_{2}=-{\vec{v}}_{1}\cdot {\vec{v}}_{2}$, which is a scalar quaternion.

Following their introduction by Hamilton, quaternions were widely used in geometry and physics until the development of vector analysis methods. While interest in quaternions is now primarily restricted to pure mathematics, they are still employed for the efficient computation of 3D rotations, as detailed in Section 1.2.

### 1.2 Quaternion representation of three-dimensional rotations

An important feature of quaternions is their ability to efficiently represent rotations in $R^{3}$ (Kuipers 1999). According to Euler’s rotation theorem, any single rotation (or sequence of rotations) in $R^{3}$ can be modeled as a rotation of angle θ about an axis (the Euler axis) that can be represented by a unit vector. Let the $R^{3}$ vector $\vec{v}$ represent a vector from the origin to the point $\left(v_{x},v_{y},v_{z}\right)$ and model $\vec{v}$ using the vector quaternion $q_{v}=v_{x}i+v_{y}j+v_{z}k$. The rotation of $\vec{v}$ by angle θ about the axis defined by unit vector $\vec{u}=(u_{x},u_{y},u_{z})$ can be represented by the rotation quaternion $r=\text{cos}(\theta /2)+\text{sin}(\theta /2)(u_{x}i+u_{y}j+u_{z}k)$ and computed mathematically as:


(6) $$q_{v}^{\prime }=rq_{v}{\left(r\right)}^{-1}$$

The coefficients of the generated vector quaternion $q_{v}^{\prime }$ capture the coordinates of the rotated vector $\vec{v}$ in $R^{3}$. The product of multiple rotation quaternions generates a quaternion whose orientation is produced by the corresponding sequence of rotations. There are several important benefits of the quaternion model for computing rotations relative to the vector analysis approach that employs a 3×3 rotation matrix:

- Rotation quaternions provide a more compact representation (4 numbers versus 9 for a rotation matrix)
- Rotation quaternions are a more interpretable representation (i.e. easy to translate between desired rotation and rotation quaternion)
- Rotation quaternions support efficient calculation of smooth rotations by robustly dividing a single large rotation into a sequence of small rotations.

These benefits have led to the frequent use of quaternions in computer graphics.

### 1.3 Analysis of biomedical data using quaternions

Given the advantages of quaternions for modeling rotations, most biological applications of quaternions have involved the structural analysis of biomolecules including problems involving protein folding (Hanson and Thakur 2012), chromatin structure (Caudai *et al.* 2015), and molecular dynamics (Miller *et al.* 2002). Outside of these structural analysis problems, use of quaternions for the analysis of biological data has been very limited with the only notable developments involving the discovery of repetitive elements within either one-dimensional sequences (e.g. DNA) or two-dimensional arrays (e.g. images). For example, Brodzik explored the use of quaternions for the detection of tandem repeats in DNA using a periodicity transformation on a novel mapping of DNA bases to quaternions (Brodzik 2007), and, in the space of image analysis, Ell and Sangwine (2007) explored the mapping of RGB pixel values into quaternions to support the analysis of color images using a two-dimensional hypercomplex Fourier transform.

### 1.4 Spatial transcriptomics

Spatial transcriptomics (ST) covers a range of technologies that profile gene expression in whole tissue samples at the resolution of single cells [e.g. MERFISH (Chen *et al.* 2015)] or small cell groups [e.g. 10× Genomics Visium technology (Ståhl *et al.* 2016)]. Although ST data has the potential to accurately characterize the biological state and functional architecture of complex tissues, statistical analysis of this data is very challenging. ST data is high-dimensional (transcript abundance is typically measured on hundreds to thousands of genes depending on the technology), extremely sparse (90% or more of gene counts are typically 0), and noisy (due to significant amplification bias and other technical factors). Given the challenges of sparsity and noise, some form of dimensionality reduction is typically employed [e.g. principal component analysis (PCA), uniform manifold approximation and projection (UMAP) (McInnes *et al.* 2018), etc.] to make subsequent statistical analyses more tractable. In particular, both clustering and visualization of ST data is usually done using a projection onto a reduced dimensional space.

Even after dimensionality reduction, a large number of variables are still associated with each profiled tissue location, which makes joint analysis of these features using spatially-aware techniques extremely challenging. In particular, application of Fourier-based techniques to ST data is typically performed via the separate analysis of each feature, i.e. a Fourier transformation is applied to a matrix whose rows and columns capture tissue coordinates and whose elements capture just a single transcriptomic variable such as expression of a specific gene. An important motivation for the work detailed in this paper is to support the joint analysis of multiple ST features using a single two-dimensional discrete Fourier transform. Note that dimensionality is discussed in both the spatial context (e.g. *x* and *y* coordinates in a tissue) and latent variable context (e.g. rank of SVD-based dimensionality reduction).

## 2 Quaternion models for ST data

A number of potential approaches exist for mapping the transcriptomic profile of ST locations to quaternions with the key limitation that a maximum of four independent dimensions can be represented. This section outlines three models that we believe have the most practical utility for ST analyses:

- Singular value decomposition (SVD) model
- Gene model
- Gene set model

Although the analysis results in the remainder of this paper are based on the SVD and gene models, the transformation of ST data according to all three models is supported by the QSC R package available at https://hrfrost.host.dartmouth.edu/QSC.

### 2.1 SVD model

Algorithm 1 SVD model

**Input:** An n×p matrix X that holds unnormalized UMI counts for *p* genes measured in *n* locations via ST.

**Output:** Length *n* vector q of quaternions that represent the projection of the *n* locations onto a rank 3 subspace of X.

**Notation:** Let $q_{a}$, $q_{b}$, $q_{c}$, and $q_{d}$ represent the real-valued coefficients for quaternion q=a+bi+cj+dk with real component *a* and vector component {b,c,d}.

 1: $X=\mathrm{UD}V^{T}$  ▹ Compute the singular value decomposition (SVD) of X

 2: $P=U_{4}D_{4}$  ▹ Project X onto the first four singular vectors of X. $U_{4}$ is a n×4 matrix that holds the first four columns of U and $D_{4}$ is a 4×4 matrix that holds the first four diagonal elements of D.

 3: $\forall_{i\in 1\ldots n}q_{i,a}=|p_{i,1}|$  ▹ Set the real component of the quaternion for location *i* to the absolute value of projection onto the first singular vector.

 4: $\forall_{i\in 1\ldots n}l_{i}=\sqrt{p_{i,2}^{2}+p_{i,3}^{2}+p_{i,4}^{2}}$  ▹ Compute the Euclidean length of the projection onto singular vectors 2, 3, and 4.

 5: $\forall_{i\in 1\ldots n}q_{i,b}=p_{i,2}/l_{i},q_{i,c}=p_{i,3}/l_{i},q_{i,d}=p_{i,4}/l_{i}$  ▹ Set the complex coefficients for the quaternion for location *i* to the coordinates of the rank 3 projection normalized to unit length.

   **return**  q

The SVD model is detailed in Algorithm 1. This approach first applies SVD to the unnormalized ST count data before mapping the projection onto the first four singular vectors to quaternions. The generated vector of quaternions can then be used to populate a quaternion matrix whose rows and columns represent horizontal and vertical tissue coordinates (see Section 4.1 below for details on how this can be performed for ST data). To improve the computational performance of Algorithm 1 on large ST data sets (e.g. 10× Visium HD or Xenium data), step 1 can be replaced by a randomized SVD algorithm (e.g. the randomized SVD implementation in the *rsvd* R package (Erichson *et al.* 2019)) with only the first four singular vectors computed. It should also be noted that comparable results can be obtained by using the projection of the ST data onto the first three principal components to create the vector quaternion and separately setting the real component of the quaternion to location library size. Alternatively, nonlinear dimensionality reduction techniques [e.g. UMAP (McInnes *et al.* 2018)] could be employed.

**Figure 1.**
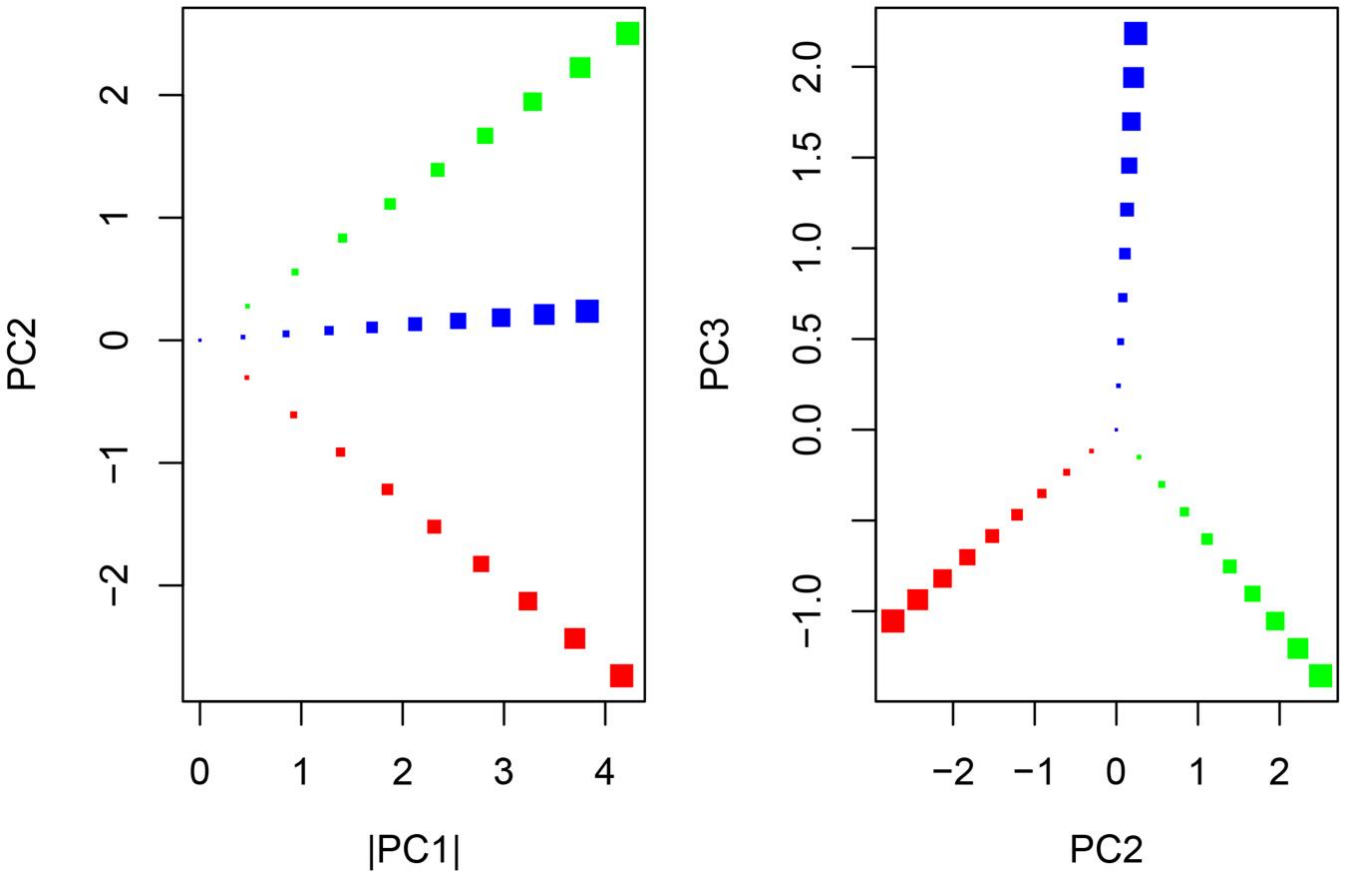
Projection of simulated ST data onto the first three singular vectors. Points with the same color represent locations with the same relative abundance profile. Point size reflects the sum of all non-relative counts for the simulated location.

For unnormalized count data, the absolute value of the projection onto the first singular vector corresponds to library size, i.e. the sum of all counts for the location, and the direction of the vector represents the reduced rank relative expression profile. Figure 1 illustrates these properties for simulated ST data for five genes and 30 locations split into three different populations. The first 10 locations were simulated with relative abundance values of {0.5,0.2,0.1,0.1,0.1}, the next 10 with relative abundance values of {0.1,0.1,0.1,0.2,0.5}, and the last 10 with relative abundance values of {0.1,0.3,0.3,0.2,0.1}. Within each population, the relative library size varied from 1 to 10. When these locations are projected onto the singular vectors, all locations sharing the same relative expression profile lie along a single line that intersects the origin with distance from the origin proportional to library size.

Given these properties, the real part of the quaternion associated with each location (as defined by Algorithm 1) represents library size, which directly corresponds to uncertainty in the estimated relative expression profile, and the vector part of the quaternion, which is normalized to unit length, represents the relative expression profile. The SVD model also has the useful property that transitions between different relative expression profiles are realized by a rotation in $R^{3}$, which can be represented by a rotation quaternion and is equivalent to the use of cosine similarity to capture the distance. Cosine similarity works well as a distance measure when the focus is on relative versus absolute values (i.e. angle will be 0 for scaled versions of the same vector so is equivalent to comparing relative data) and is therefore appropriate for ST data that is typically normalized by library size to generate relative values.

### 2.2 Gene and gene set models

Instead of using the latent variables generated using SVD or another dimensionality reduction technique, the sample-level expression of individual genes or gene sets can be mapped to the quaternion coefficients. For example, a location-level gene set scoring method such as our Variance-adjusted Mahalanobois (Frost 2020) or Reconstruction Set Test (Frost 2024) methods could be used to score three distinct gene sets (e.g. gene sets that capture different types of cytokine signaling, or gene sets that represent different cell type signatures) at each location and these scores could then be used for coefficients of the vector part of the quaternion with the real part representing total sequencing depth for the associated genes. An interesting special case of a cell type-based mapping would use the proportions for three cell types (or groups of cell types) generated by a deconvolution method such as Cell2location (Kleshchevnikov *et al.* 2022).

Alternatively, the mapping could be performed on the expression levels of three individual genes (either the normalized expression values or the reduced rank reconstructed values). While a gene or gene set model only considers a subset of the expression profile, it has the benefit of a more straightforward biological interpretation and a more comprehensive analysis could be realized via multiple quaternion mappings on different collections of genes or gene sets. Similar to the SVD model, the gene and gene set quaternion models have the benefits of (i) capturing expression uncertainty using the real component of the quaternion, and (ii) representing transitions between different relative expression profiles using rotations in $R^{3}$.

## 3 Quaternion model applications

This section details the three primary applications of quaternion model explored in this article: visualization of ST data, rotation-based analysis of ST data, and Fourier-based analysis of ST data. It is important to note that the first two applications, while more efficiently/elegantly realized using quaternions, do not strictly require a quaternion mapping, i.e. they can be realized by the vector analysis of the underlying transcriptomic variables. The last application, however, can only be fully realized by a quaternion representation of the transcriptomic data. Additional applications, e.g. visualization of cell state uncertainty, characterization of cell state transitions, and modeling of scRNA-seq data, are detailed in the Section 6 at the end of the manuscript.

### 3.1 Visualization of ST data

Single-cell data is commonly visualized via a projection onto the top two reduced dimensions computed using techniques such as PCA or UMAP, However, this type of two-dimensional visualization cannot be applied to ST data without losing spatial context. Visualization of ST data that preserves the spatial tissue structure is therefore usually performed for just a single gene at a time with color representing expression magnitude. Our proposed quaternion model enables a range of false color visualizations that capture both tissue architecture and a representation of the entire transcriptional profile. Specifically, the four real coefficients associated with each quaternion, (a,b,c,d), can be mapped into red, green, blue (RGB) and α (i.e. opacity) values. One obvious mapping, which was employed by Sangwine (1998) and Ell and Sangwine (2007) to map from RGB values to quaternions sets the imaginary quaternion coefficients *b*, *c*, *d* to the red, green and blue values (see Ell and Sangwine 2007, for more details on the utility of this mapping for various image analysis tasks). We extend that mapping to also use the real quaternion coefficient *a* to represent the α opacity value. This mapping has the benefit that the relative transcriptional profile of each ST location corresponds to a color with the opacity based on the library size. Specifically, locations with no reads will be fully transparent and those with the most reads, and therefore the least uncertain expression profiles, will be fully opaque.

To support this mapping, the quaternion *a*, *b*, *c*, and *d* coefficients must be transformed into the 0-to-1 range. For the imaginary *b*, *c*, and *d* coefficients (which are bound between −1 and 1 since the vector component has unit length) the transformation is realized using the sigmoid function f(x)=1/(1+exp(−2b). The quaternion *a* coefficient is positive and we have found that effective opacity α values can be generated by first thresholding all values at the 0.9 quantile, then applying the log-transformation log(x−min(x)+1) and finally linearly rescaling to the 0-to-1 range.

### 3.2 Rotation-based analysis of ST data

One of the key features of the proposed quaternion model is that the relative expression profile of each location is represented by a vector in $R^{3}$. This representation implies that transitions between the transcriptomic states of different locations is realized by a rotation in $R^{3}$, which can be represented by a rotation quaternion. Importantly, the rotation axis defined by the rotation quaternion corresponds to a transcriptomic state and thus provides a biological interpretation for the associated state transition. The rotation axis also represents the state that is invariant to the associated transition, i.e. a tissue location in that state can be interpreted as being unaffected by whatever biological perturbation triggers the state transition. Although viewing cell state transitions in terms of an invariant or orthogonal state may not always be intuitive, it does provide a unique biological perspective on transcriptomic state differences. Rotation-based analyses that leverage these properties include:

- *Revealing tissue locations that are invariant to a particular biological perturbation.* If a perturbation of interest can be defined as the transition between two relative expression profiles (e.g. the expression profiles represented by two locations in an ST dataset), then the associated rotation can be applied to an entire ST dataset to help identify locations that are potentially invariant to the perturbation. Perturbation invariance can be highlighted by visualizing the difference between the original quaternion matrix and the matrix generated by the rotation. In the scenario where both normal and perturbed ST datasets are available, the rotation quaternion associated with a perturbation could be identified, validated and applied using the following procedure:
  - Identify the relevant subset of spots/cells in each ST dataset, e.g. all CD8+ T cells, all cells in a given anatomical region, etc.
  - Generate a quaternion mapping for each ST dataset and compute the average quaternion for the spots/cells in the target subset within each dataset.
  - The rotation representing the perturbation is represented by the rotation needed to transform the average quaternion for the control spots into the average for the perturbed spots.
  - To assess how well the perturbation can be modeled by a rotation, rotation quaternions can be computed between all pairs (or a random subset of pairs) of control/perturbation spots. If there is a large variance among the pairwise rotation quaternions, the perturbation is unlikely to be effectively modeled by a single rotation.
  - If a stable perturbation rotation is found, it can be applied to the relevant spots in another normal condition ST dataset to predict the impact of the perturbation.
- *Identifying multivariate associations.* To identify associations between the transcriptomic features captured by the vector part of the quaternion for each location, a rotation can be applied about an axis that models the association of interest. For example, for a gene-based quaternion model, a positive association between all three genes could be identified by applying a rotation about the axis represented by the quaternion q=i+j+k. Locations where the expression of the three genes is all positive (or all negative), will be invariant to the rotation and can be identified by visualizing the difference between the unrotated and rotated quaternion ST matrices.

### 3.3 Spectral analysis of ST data using hypercomplex Fourier transformation

The genome-wide spectral analysis of ST data is challenging. For exploring the spectra of ST data in two dimensions (e.g. spatial location), a two-dimensional discrete Fourier analysis is typically performed on the expression values for each gene separately. Alternatively, the projection of each location onto a reduced dimension (e.g. PC or UMAP dimension) or pathway-based aggregation could be used but exploring the joint spectral characteristics of multiple gene expression variables is challenging. Ell and Sangwine (2007) used a mapping from RGB color values to quaternions to support the application of a two-dimensional quaternion-domain discrete Fourier transform (Hitzer 2016) to color image data. As detailed by Ell and Sangwine, this quaternion-domain Fourier analysis can capture image features associated with interactions between the components that would be challenging to detect using separate Fourier transformations of each color component. Our proposed quaternion model enables the spectral analysis of a multivariate representation of ST data relative to spatial location via a two-dimensional quaternion-domain discrete Fourier transformation applied to the quaternion representations of each location. As compared to separate Fourier transforms each individual feature (gene or latent variable), the quaternion model can capture the joint distribution, i.e. coepxression structure. The output from such a two-dimensional discrete Fourier transform has a wide range of important applications (Jain 1989), including spectral filtering, convolution-based analyses, matrix reconstruction (John *et al.* 2020), and registration of multiple matrices (Tong *et al.* 2019). Examples of spectral filtering and convolution for ST data are explored for a mouse brain Visium HD dataset in the sections below.

To realize this quaternion-domain Fourier transform using standard complex-domain discrete Fourier transform implementations, we can follow the approach of Ell and Sangwine (2007):

1. Rewrite the quaternions in the input matrix Q (for ST data) in Cayley–Dickson form:
  (7) q=a+bi+cj+dk=α+βj with α=a+bi,β=c+di
2. Create two new complex-values matrices ($C_{\alpha },C_{\beta }$) that hold the α and β values from the Cayley–Dickson representation.
3. Compute the two-dimensional complex-domain discrete Fourier transforms of $C_{\alpha },C_{\beta }$. This can be realized using standard FFT implementations such as the *fft()* function in R. Let the output from these Fourier transforms be held in complex-valued matrices $F_{\alpha },F_{\beta }$.
4. Construct the quaternion-valued DFT output matrix F using the outputs of the complex-domain Fourier transform, e.g. $F=F_{\alpha }+F_{\beta }j$.

## 4 Analysis of mouse brain ST data

**Figure 2.**
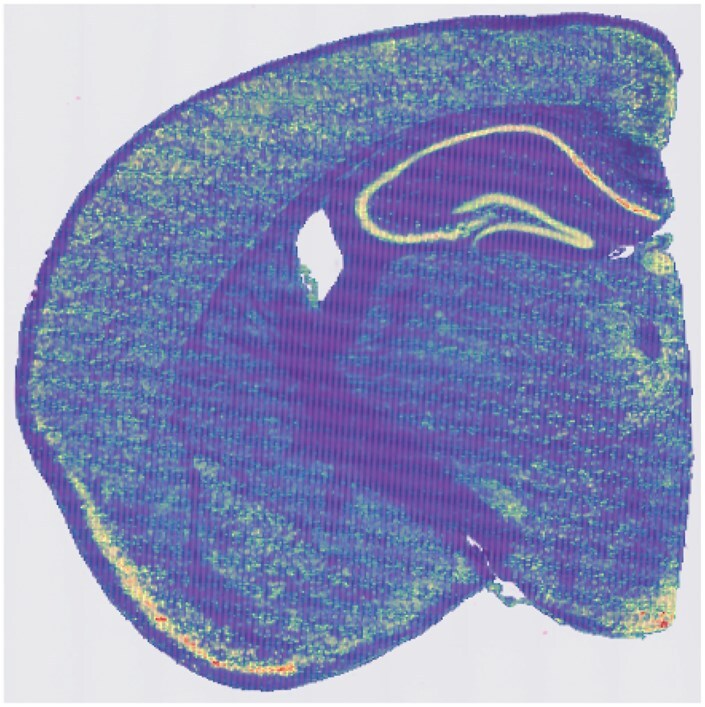
Visualization of 10× Visium HD spatial transcriptomics data for coronal mouse brain slice. The H&E stained image is displayed behind a visualization of the Visium spots colored according to the number of UMI counts.

To illustrate the proposed quaternion model and the ST visualization, rotation and hypercomplex Fourier analysis applications, we analysed a 10× Visium HD ST dataset generated on a mouse coronal brain slice (results for a mouse brain sagittal Visium dataset, a mouse kidney Visium dataset, and human ovarian cancer Vizgen MERSCOPE dataset are included as vingettes with the QSC R package). This particular dataset is accessible via 10× at https://www.10xgenomics.com/datasets/visium-hd-cytassist-gene-expression-libraries-of-mouse-brain-he. When analyzed at the 16 μm resolution, this Visium HD dataset has 98 917 non-empty locations. Figure 2 shows the H&E stained section overlaid by a visualization of the Visium grid with color corresponding to the number of detected UMI counts. Processing of this ST data was realized using the Seurat framework (Butler *et al.* 2018) and QSC R package (available at https://hrfrost.host.dartmouth.edu/QSC). The QSC package implements the proposed quaternion models using the hypercomplex number functionality in the *onion* R package (Hankin 2021) and randomized SVD functionality in the *rsvd* R package (Erichson *et al.* 2019). Other important functions implemented by the QSC package include the quaternion-valued discrete Fourier transform (realized using the Cayley–Dickson approach detailed in Section 3.3), Fourier-based convolution of quaternion-valued matrices, generation and application of rotation quaternions, false color visualization of quaternion-valued matrices (using the approach in Section 3.1), and mapping of ST data to quaternion matrices (see section below).

### 4.1 Mapping of mouse brain ST data to quaternion matrix

**Figure 3.**
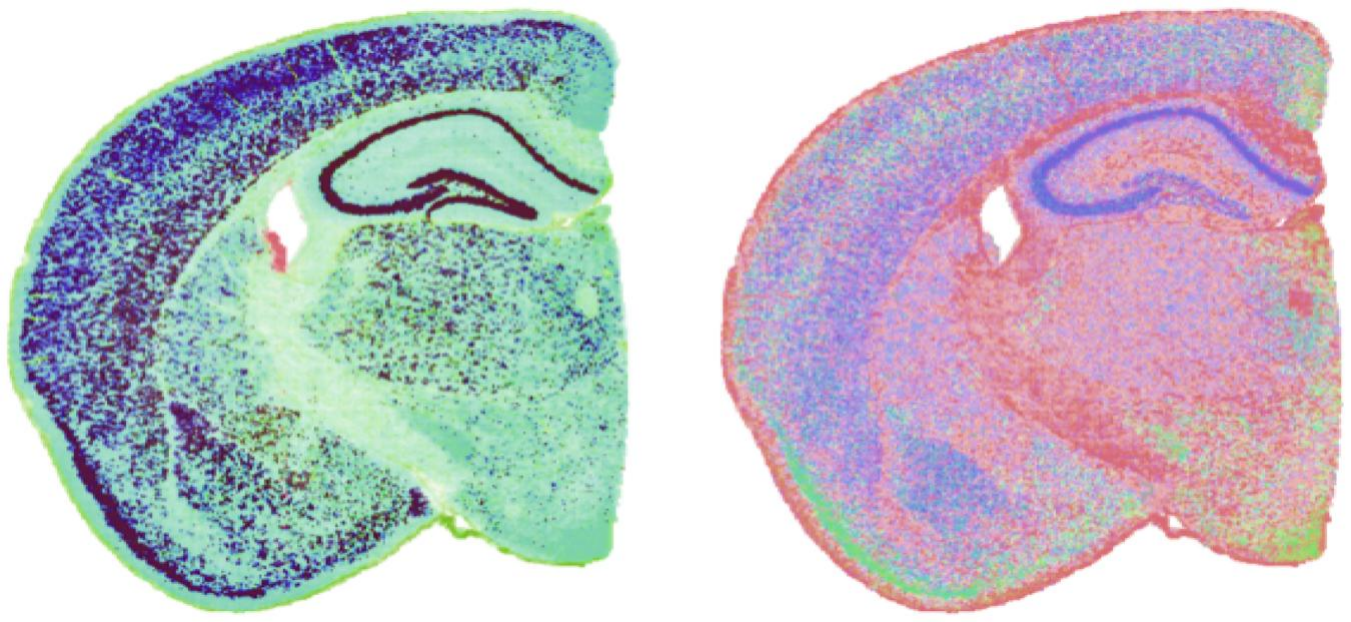
Visualization of quaternion mapping for coronal mouse brain slice Visium HD data. The left panel shows the SVD model and the right panel shows a mapping based on the rank 20 reconstructed expression of the GFAP, Reln, and Neurod6 genes, which are markers for astrocytes, interneurons and excitatory neurons and are mapped to red, green, and blue respectively.

Quaternion matrices for the mouse brain ST data were generated using both the SVD and gene-based models. For the gene-based model, the *i*, *j*, and *k* vector components of each quaternion were set to the expression of the rank 20 reconstructed expression of GFAP, Reln, and Neurod6 genes, which are markers for astrocytes, interneurons, and excitatory neurons, respectively (i.e. locations with a high proportion of astrocytes will appear red, those associated with interneurons green and those associated with excitatory neurons blue). The quaternion values for each location were then used to populate 368 × 465 matrices whose rows represent evenly spaced vertical coordinates and whose columns represent evenly spaced horizontal coordinates on the Visium slide. For matrix elements not mapping to a spot, the quaternion value was initially set to (0,0,0,0). Given alignment/rotation issues between the x, y coordinates and the Visium slide, some matrix elements within the tissue did not map to a Visium location and a simple fill-in strategy was used that replaced empty elements with the average of adjacent non-zero values. The resulting quaternion matrix representations of the mouse brain Visium HD data is visualized in Fig. 3 using the false color mapping detailed in the Section 3.1.

### 4.2 Rotation of mouse brain quaternion matrix

**Figure 4.**
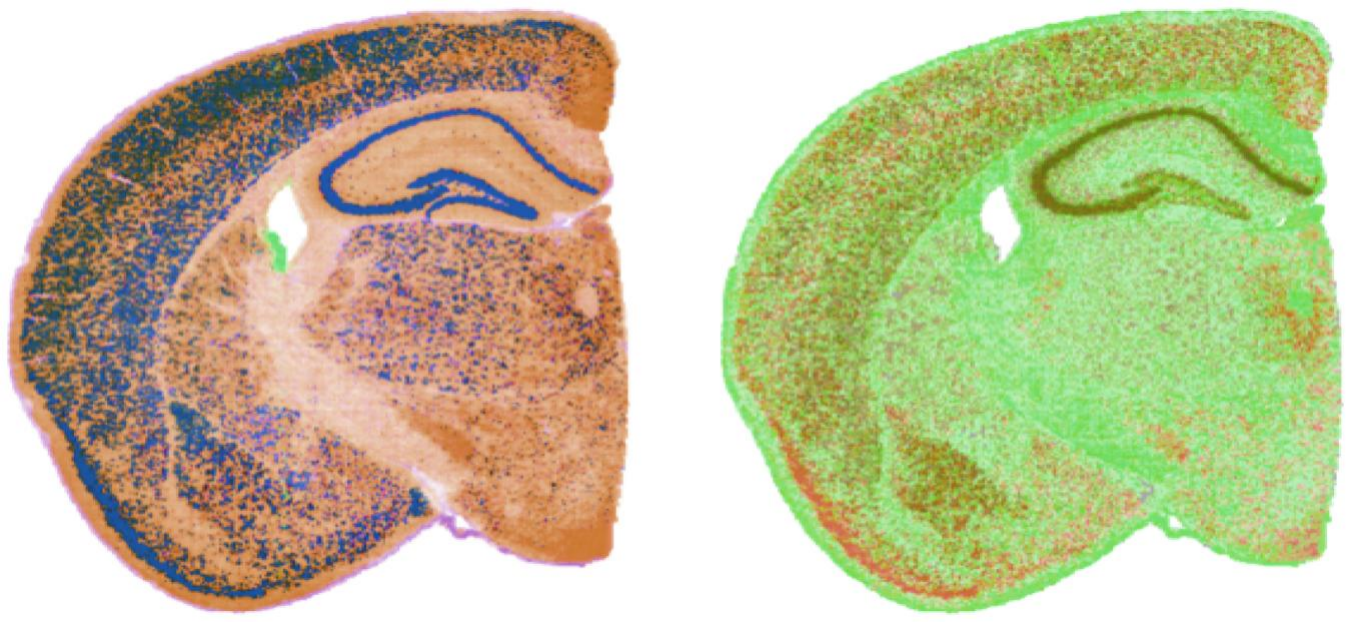
Visualization of the mouse brain quaternion matrix rotations. Left panel shows the 180° rotation SVD-based quaternion matrix about the i+j quaternion. Right panel shows the rotation of the gene-based quaternion matrix about the i+j quaternion.

An important benefit of the proposed quaternion model is that the vector part of each quaternion captures the relative expression profile of the associated cell/location and can be interpreted as a vector in $R^{3}$. As detailed above, the primary practical application of quaternions is the efficient computation of 3D rotations. Figure 4 visualizes rotations of the SVD (left) and gene-based (right) quaternion representations of the mouse brain ST data. Both panels display the result of rotating the vector components of each quaternion by 180° about the i+j quaternion axis. This results in changes to the quaternion for each location based on the similarity between the expression profile for that location and this axis. For the SVD-based model, this will result in large changes to locations with small projections on the second and third singular vectors and a large projection on the fourth singular vector. For the gene-based model, this will result in large changes to locations with low reconstructed expression of GFAP and Reln and large reconstructed expression of Neurod6.

**Figure 5.**
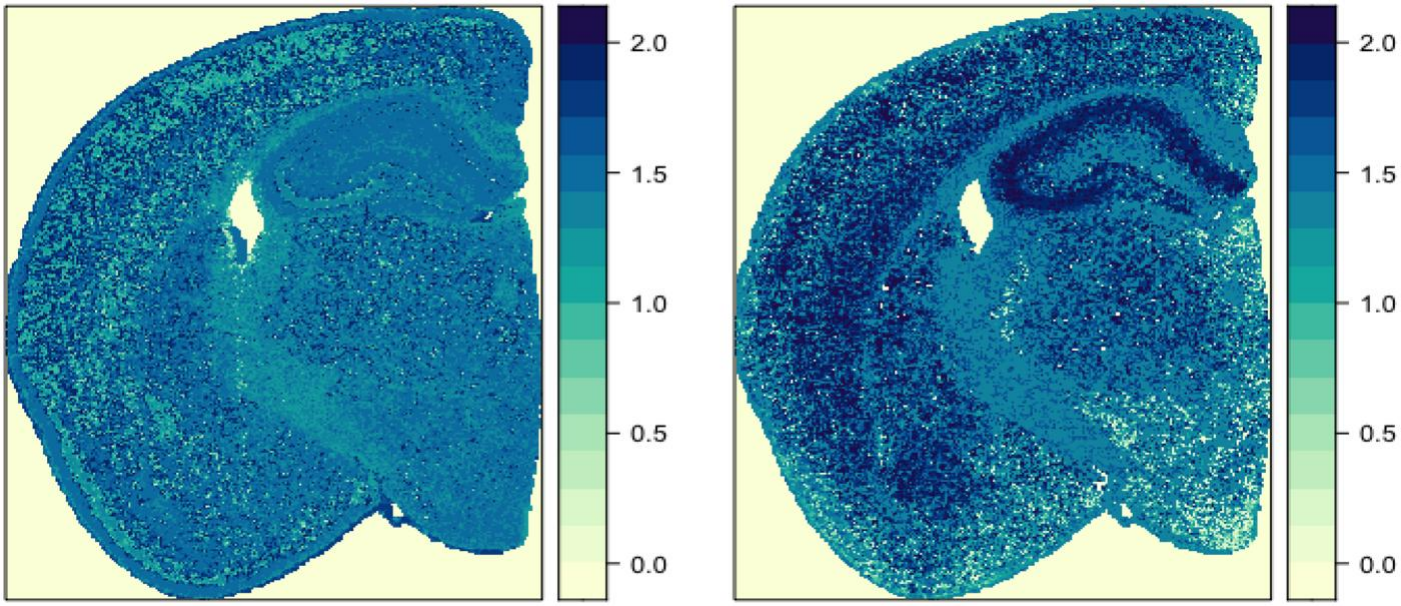
Visualization of the length of the vector quaternion portion of the difference between the unrotated and rotated mouse brain quaternion matrix. Darker color indicates locations with a relative expression profile whose corresponding vector quaternion is orthogonal to the i+j axis. Left panel visualizes the results for the SVD-based quaternion matrix about the right panel shows the results for the gene-based quaternion matrix.

While the false color representation of the rotated quaternion matrices is visually striking, it can be challenging to interpret. To aid in interpretation of rotation-based analyses, one can instead visualize the Euclidean length of the vector quaternions generated by taking the difference between the original and rotated quaternion matrices. In particular, if the original vector quaternion is similar to the rotation axis, the elements in the difference matrix will have a small length. In contrast, if the original vector quaternion is approximately orthogonal to the rotation axis, the elements in the difference matrix will have lengths that are close to 1 (the length of all vector quaternions in the original matrix). A visualization of these difference lengths for both the SVD and gene-based models given the 180° rotation about i+j is shown in Fig. 5. For the gene-based model (right panel), this type of visualization makes it very clear which locations are dominated by astrocytes and interneurons (light color corresponding to short lengths) or are dominated by just excitatory neurons (dark color corresponding to a length close to 1). In general, this technique can enable users to clearly visualize deviation from a target relative expression profile.

### 4.3 Spectral filtering of mouse brain quaternion matrix

**Figure 6.**
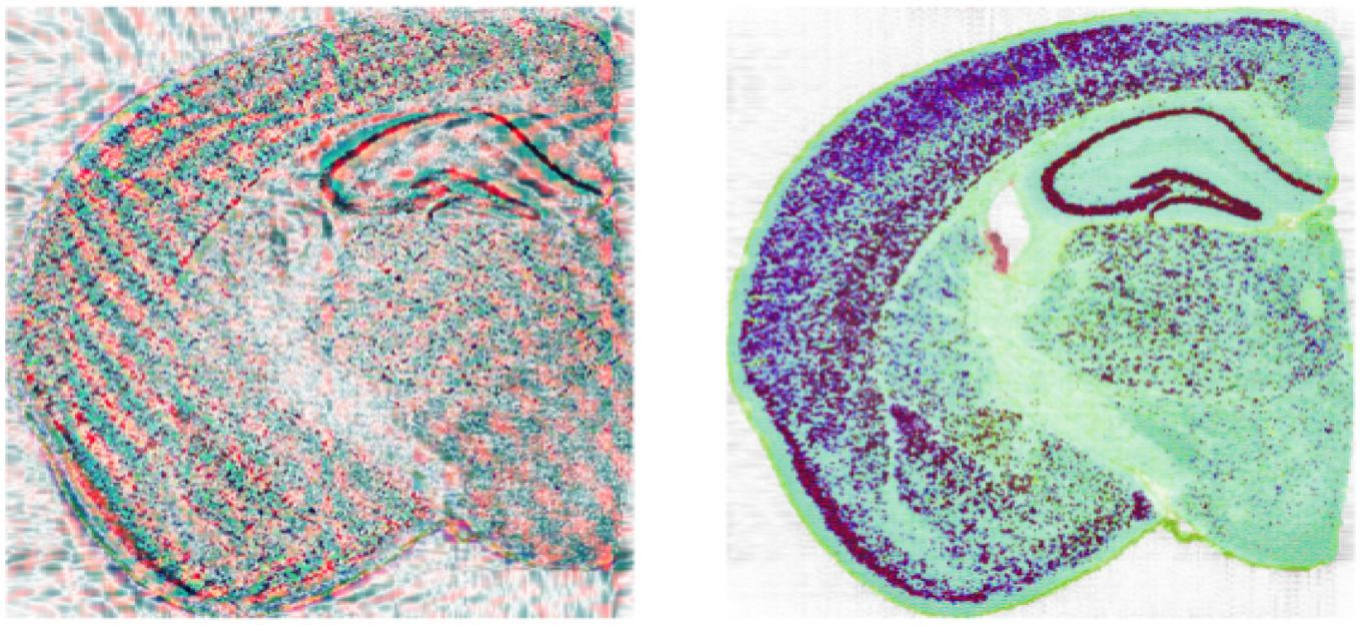
Output of low-pass (left panel) or high-pass (right panel) filtering of the SVD-based mouse brain quaternion matrix.

The proposed quaternion model enables the spectral analysis of ST data using a quaternion-domain two-dimensional discrete Fourier transform. Filtering (or other operations) performed on the result of this transformation are computed on the joint spectral representation of the projection onto the first four singular vectors of the unnormalized data. Importantly, this type of joint analysis cannot be directly replicated via real-domain two-dimensional discrete Fourier transforms applied to each dimension separately. One type of simple Fourier-based analysis involves spectral filtering to remove target frequency components. Specifically, the quaternion representation of ST data is transformed into the frequency space using a quaternion-domain discrete Fourier transform (as detailed in Section 3.3), the quaternion values for undesired frequency components are set to 0 and result is transformed back using an inverse quaternion-domain discrete Fourier transform. The results generated by this type of spectral filtering of the SVD-based mouse brain quaternion matrix are visualized in Fig. 6 with the left panel displaying the output of low-pass filtering that removes the top 10 high frequency components and the right panel displaying the output of high-pass filtering that removes the bottom 10 low frequency components. The banding pattern seen after low-pass filtering is due to the structure of the Visium HD x, y coordinates for this dataset.

### 4.4 Convolution of mouse brain quaternion matrix with real-valued mask

The most important use case for quaternion-domain Fourier analysis is the computation of convolutions. Convolving image data with appropriate mask matrices is a critical step in many imaging processing pipelines and the QSC package provides support for the convolution of quaternion-valued matrices and generation of mask matrices that embed real or quaternion-valued kernels. For the convolution of matrices generated according to the proposed quaternion model, the real component of the quaternions is ignored and the convolution is only applied to the vector portion that represents the relative transcriptomic profile.


(8) $$\begin{array}{c} K_{\mathrm{sharp}}=\left[\begin{array}{ccc} 0 & -1 & 0\\ -1 & 5 & -1\\ 0 & -1 & 0 \end{array}\right],K_{\mathrm{edge}}=\left[\begin{array}{ccc} -1 & -1 & -1\\ -1 & 8 & -1\\ -1 & -1 & -1 \end{array}\right] \end{array}$$

**Figure 7.**
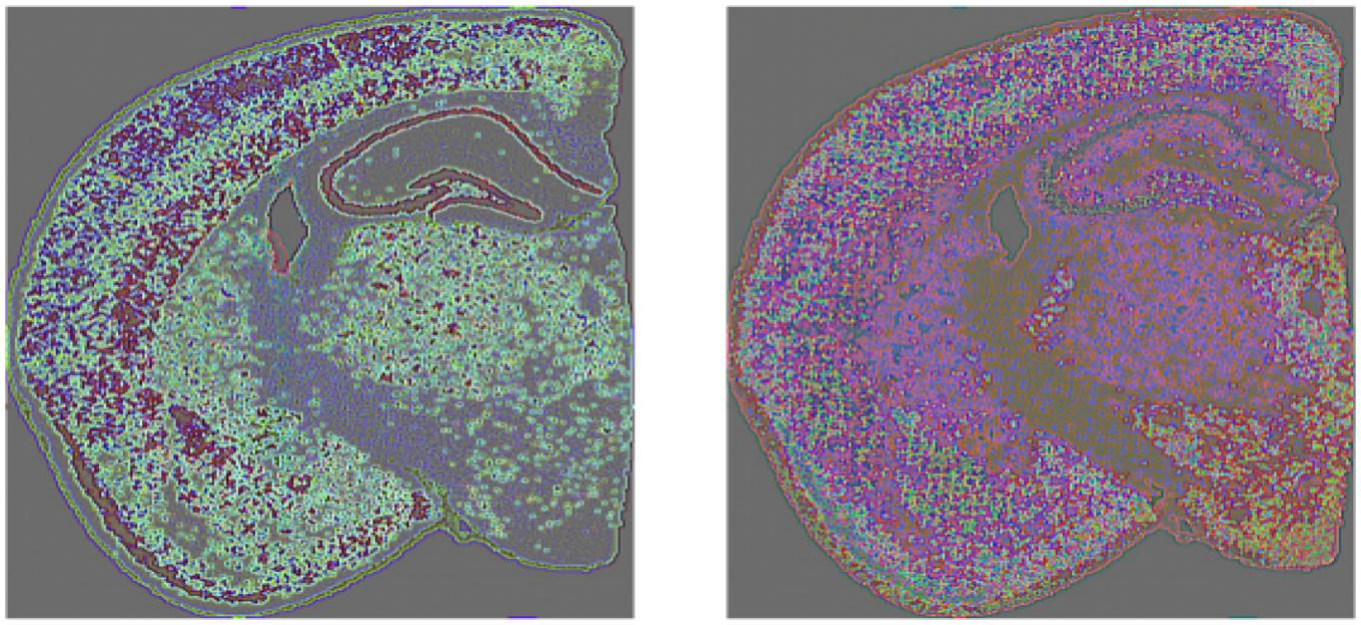
Convolution of mouse brain quaternion matrix with edge detection kernel. Left panel shows the output for the SVD-based quaternion matrix and the right panel shows the output for the gene-based quaternion matrix.

Figure 7 illustrates the output from the convolution of the quaternion matrix representations of the mouse brain ST data with a real-valued mask matrix that embeds the 3 × 3 edge detection kernel $K_{\mathrm{edge}}$ (opacity is ignored to improve edge visualization). The left panel shows the results of this edge detection convolution for the SVD-based quaternion matrix and the right panel shows the results for the gene-based quaternion matrix. These edges capture the boundaries between the major tissue regions captured by either the top singular vectors or the GFAP, Reln, and Neurod6 genes (i.e. boundaries between brain regions with different mixtures of astrocytes, interneurons, and excitatory neurons).

### 4.5 Biconvolution of mouse brain quaternion matrix with quaternion-valued masks

Although real-valued mask matrices that embed standard kernels (e.g. $K_{\mathrm{sharp}}$ and $K_{\mathrm{edge}}$) can be used, the true power of the quaternion model is realized by convolution of a quaternion-valued matrix representing an ST dataset with a quaternion-valued mask matrix. However, convolution with a quaternion-valued mask matrix encounters two complications:

- Quaternion multiplication is non-commutative, so the left and right convolutions yield different results.
- Multiplication of two vector quaternions generates a quaternion with a real component.

Given these complications, design of a useful single quaternion convolution mask matrix is challenging. These complications can be avoided through the biconvolution of a matrix of vector quaternions (X) by left ($M_{L}$) and right ($M_{R}$) mask matrices:


(9) $$M_{L}*X*M_{R}$$

This type of structure has particular relevance in this context since rotation of a vector quaternion *q* is realized by pre and post-multiplication by a rotation quaternion *r* and its inverse via $rqr^{-1}$. As explored by Ell and Sangwine (2007), a version of edge detection can be performing via biconvolution with mask matrices that embed the following 3 × 3 left and right quaternion-valued kernel matrices where *r* is a rotation quaternion:


(10) $$\begin{array}{c} K_{L}=\left[\begin{array}{ccc} r & 0 & r^{-1}\\ r & 0 & r^{-1}\\ r & 0 & r^{-1} \end{array}\right],K_{R}=\left[\begin{array}{ccc} r^{-1} & 0 & r\\ r^{-1} & 0 & r\\ r^{-1} & 0 & r \end{array}\right] \end{array}$$

**Figure 8.**
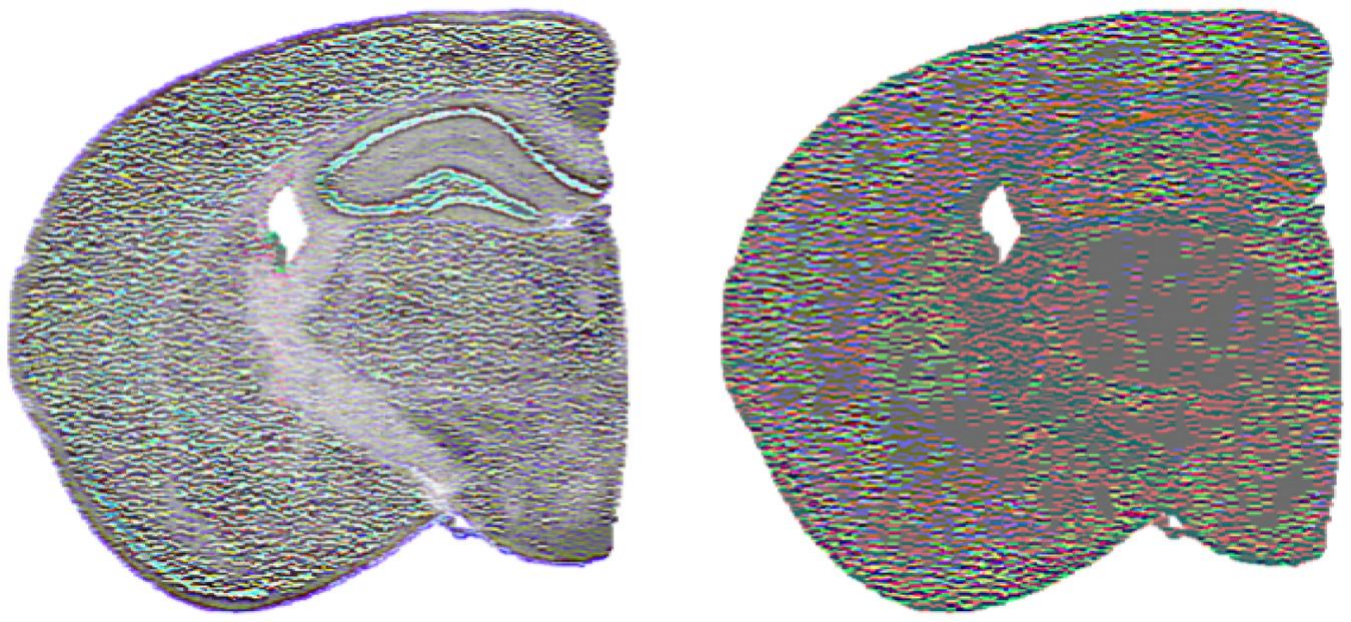
Biconvolution of mouse brain quaternion matrix with quaternion-valued kernels that perform edge detection via rotation about the *i* quaternion unit vector. The left panel shows the output for the SVD-based quaternion matrix and the right panel shows the output for the gene-based quaternion matrix.

Biconvolution with these kernels will perform edge detection in the space orthogonal to the rotation axis, i.e. changes in the direction of the rotation axis will be ignored and changes in directions orthogonal to the rotation axis will be highlighted. A straightforward example illustrated in Fig. 8 sets *r* to a 180° rotation about the *i* axis, which will detect transitions in the *j* and *k* components. The left panel shows the output of this biconvolution for the SVD-based quaternion matrix (i.e. highlights transitions in the projections of the locations onto the second and third singular vectors) and the right panel shows the output for this biconvolution for the gene-based quaternion matrix (i.e. highlights transitions in the relative abundance of interneurons and excitatory neurons). According to the false color visualization, these will be seen as edges in the green and blue portions of the spectrum.

## 5 Limitations

There are three important limitations of our proposed quaternion model.

1. The mapping from ST data to quaternions detailed in Algorithm 1 only uses the information in a rank 3 reduction of the unnormalized data, which will ignore a potentially substantial portion of the biological signal. It is important to note in this context that the typical visualization/interpretation of scRNA-seq and ST data involves a two-dimensional projection, i.e. projection of the cells/locations onto the first two UMAP dimensions, so significant utility can still be obtained from our model even if it misses biologically relevant features. In particular, the quaternion model should effectively capture distinctions between cell types/subtypes for ST data with single-cell resolution and between major tissue domains for lower resolution ST data. Potential approaches for addressing this limitation include the analysis of multiple quaternion mappings that capture a broader range of singular vectors or multiple groups of genes and exploration of mapping to octonions. It is also worth noting that the biological interpretation of rotations as cell state transitions explored in this article is relevant in a non-quaternion context to an arbitrarily large latent space model.
2. The non-commutative nature of quaternion multiplication makes the design and interpretation of techniques like convolution challenging. As illustrated through the ST example above, this challenge is mitigated by interpreting pure quaternions as vectors in $R^{3}$ and focusing on rotation-based operations.
3. Comparative evaluation of the model relative to other ST analysis techniques is challenging. The results in the current paper are largely illustrative/descriptive. This evaluation challenge is driven by a number of factors including the fact that many features of a quaternion model do not have a direct analog among other models/methods, and the fact that most ST datasets lack ground truth information.

A more minor limitation of this approach is related to computational performance. QSC R package leverages the onion R package for quaternion support and, although operations on quaternion matrices via the *onion* implementation are not as fast as those on standard matrices, computational time is still tractable. The most expensive step is creating the quaternion matrix from input ST data; however, that only takes 2 mins on a standard laptop for the example mouse brain Visium HD dataset. Analysis operations such as convolution via quaternion-domain Fourier transforms only take a few seconds on a standard laptop for the Visium HD dataset. This efficiency is due to the fact that the quaternion-domain Fourier transform can be realized in terms of complex-domain transforms, which have a very efficient FFT-based implementation for the discrete case.

## 6. Future directions

In future work, we plan to address the limitations detailed above and a number of new applications (quaternion modeling of scRNA-seq data, visualization of transcriptomic uncertainty, and characterization of cell state transitions in pseudotemporal ordering) that are outlined in the following sections. To address the first limitation, we plan to explore the joint analysis of multiple quaternion models that capture distinct groups of singular vectors, genes, or gene sets. To address the second limitation, we will design quaternion-valued convolution masks that are not rotation-based and can be used without biconvolution (e.g. masks that use the inverse of target quaternions in the kernel). For the third limitation, we plan to evaluate the rotation and Fourier-based analyses using a combination of ST simulation models (which will allow for a clear ground truth) and real ST data with biological annotations. Specifically, we will explore mapping the quaternion matrix output via rotations or Fourier analysis back into a four-dimensional latent space for use with standard ST analysis techniques such as clustering for tissue domain detection and spatial auto-correlation. We will also compare the results that can be generated via the quaternion model against the outputs from similar techniques (e.g. separate Fourier transforms on each reduced dimension, or output from tensor-based methods).

### 6.1 Quaternion modeling of single-cell RNA-sequencing data

Although the focus of this manuscript is ST data, the same quaternion modeling approach can be applied to other forms of bulk or single-cell count-based data, e.g. single-cell RNA-sequencing (scRNA-seq). For scRNA-seq data, the three quaternion modeling approaches detailed above can be applied directly, i.e. instead of mapping each ST location to a quaternion, each cell is mapped to a quaternion. The primary motivation for representing scRNA-seq data using quaternions is to support the analysis of cell ordering (e.g. pseudotemporal or lineage ordering) via a one-dimensional quaternion-domain discrete Fourier transform (see Section 6.3 below).

### 6.2 Visualization of transcriptomic state uncertainty

ST and scRNA-seq data is commonly visualized by projecting normalized locations/cells onto a two-dimensional subspace with the first two PCs or first two UMAP dimensions typical options. Although library size-based normalization does enable the comparative evaluation of cells/locations that may contain different amounts of mRNA or have different capture efficiencies/sequencing depths, it introduces an important limitation for standard visualization techniques (and other statistical analyses). Specifically, the uncertainty in the relative abundance values generated by normalization is inversely proportional to library size, however, this information is lost after normalization. Our quaternion modeling approach represents ST locations (or cells if applied to scRNA-seq) as vectors in $R^{3}$ and therefore enables a visualization that captures both the data characteristics seen in a two-dimensional projection of normalized data as well as uncertainty in the relative expression profile. Specifically, all cells/locations that share a similar relative expression profile will be distributed along a line that intersects the origin with the distance from the origin capturing relative library size. Cells/locations far from the origin therefore have a much more accurately inferred state than those close to the origin. This property can be used informally by users to guide the interpretation of the visualized scRNA-seq/ST data or formally to generate confidence regions for each cell/location according to a specific statistical model for the unnormalized counts.

### 6.3 Characterization of cell state transitions in pseudotemporal ordering

The proposed quaternion model has the important implication that transitions between the transcriptomic states is realized by a rotation in $R^{3}$, which can be represented by a rotation quaternion. One potential application of this transition model involves the interpretation of trajectories generated by pseudotemporal ordering methods such as Monocle (Qiu *et al.* 2017) (or other cell orderings such as those created by lineage tracing techniques). Specifically, the transitions between cells lying on a computed trajectory correspond to rotations and therefore have an interpretation via the associated invariant cell state. The quaternion rotation model also makes it possible to efficiently compute over the set of potential paths that connect cells in distal parts of the trajectory, e.g. cell at the root and cells at the leaf nodes, which can be utilized to estimate the likelihood of the observed trajectory relative to the space of possible trajectories.

## 7 Conclusion

Quaternions are four-dimensional hypercomplex numbers that have been primarily leveraged to represent three-dimensional rotations. In this article, we detail a novel approach for mapping ST data to quaternions. According to this model, the quaternion associated with each ST location represents a vector in $R^{3}$ with vector length capturing sequencing depth and vector direction capturing the relative expression profile. This approach supports a number of applications including the visualization of cell state uncertainty, characterization of cell state transitions, visualization of ST data and, most importantly, the Fourier-based analysis of ST data. To demonstrate the feasibility of this approach, we mapped ST data for both Visium and Vizgen ST datasets to matrices of quaternions and explored a number of downstream analyses including the use of a quaternion-domain two-dimensional discrete Fourier transform to compute convolutions and biconvolutions of the ST quaternion matrix with real and quaternion-valued mask matrices. An R package supporting our proposed model and the hypercomplex Fourier analysis of ST data along with several example vignettes is available at https://hrfrost.host.dartmouth.edu/QSC.

## Data Availability

An R package supporting our proposed model and the hypercomplex Fourier analysis of ST data along is available at https://hrfrost.host.dartmouth.edu/QSC/QSC_0.7.0.tar.gz. The mouse brain Visium HD spatial transcriptomics data used to generate the results in the paper is available from 10x at https://www.10xgenomics.com/datasets/visium-hd-cytassist-gene-expression-libraries-of-mouse-brain-he. The mouse brain Visium spatial transcriptomics data used to generate the vignette in the QSC R package is accessible via the SeuratData R package as the *stxBrain* dataset. The mouse kidney Visium spatial transcriptomics data used to generate the supplemental vignette QSC_VisiumAnalysis_MouseKidney.pdf is accessible via the SeuratData R package as the *stxKidney* dataset. The human ovarian cancer Vizgen spatial transcriptomics data used to generate the supplemental vignette QSC_VizgenAnalysis_OvarianCancer.pdf is available from the MERSCOPE FFPE is available from the MERSCOPE FFPE Human Immuno-oncology Data Release provided by Vizgen (https://vizgen.com/data-release-program/).
